# Supplementary material for: Frequent Anti-V1V2 Responses Induced by HIV-DNA Followed by HIV-MVA with or without CN54rgp140/GLA-AF in Healthy African Volunteers
Source: Microorganisms. 2020 Nov 4;8(11):1722. doi: 10.3390/microorganisms8111722 (PMC7693996; doi:10.3390/microorganisms8111722)
Supplement: Supplementary file 1 [file microorganisms-08-01722-s001.zip › Supplemetal materials/Supplemental Fig 1.docx]

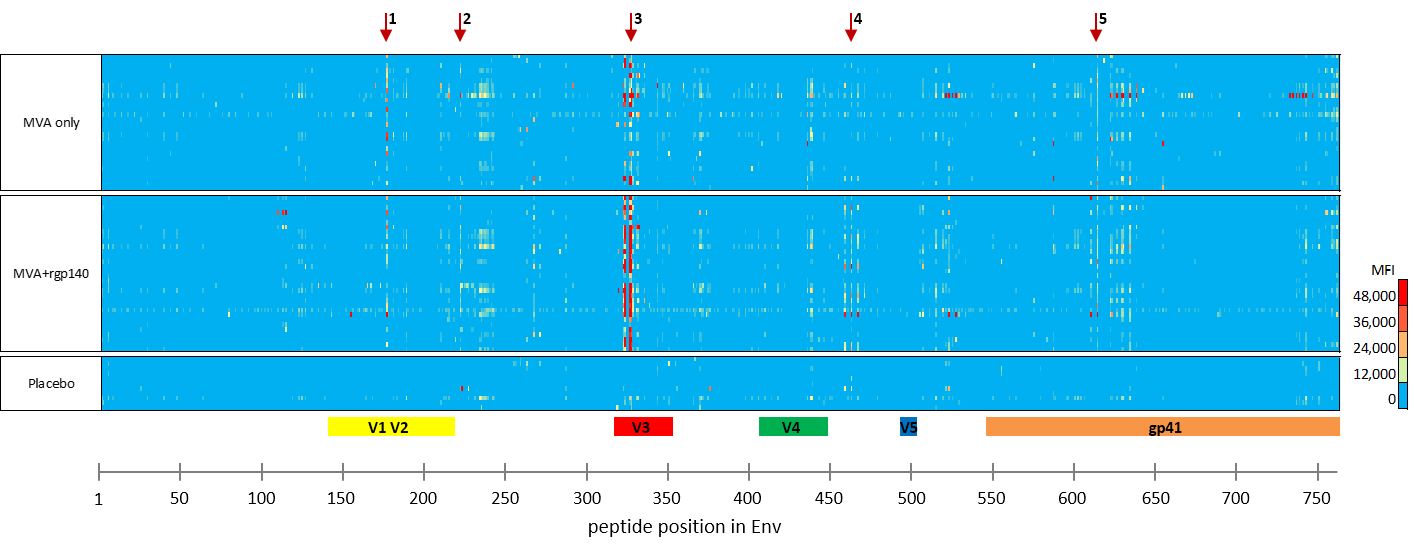


**Supplemental Figure 1:** Heat map of maximum fluorescence intensity (FI) of each vaccinee plotted against individual antigenic regions along the entire HIV-1 Env as included in the peptide microarray four weeks after the last vaccination. Each row represents one vaccinee (HIV-MVA only n = 28; HIV-MVA + CN54rgp140/GLA-AF n = 32 or placebo (n = 11). FI values corresponding to each peptide were mapped to the 10 full-length Env sequences included in the peptide array (HIV primary isolates subtypes A, B, C, CRF01_AE and CRF02_AG and HIV vaccines HIV-MVA (CRF01_AE) and CN54rgp140 (subtype C)). Responses above 2500 FI after baseline subtraction were considered positive and the maximum FI was selected per position. Immuno-dominant regions (IDRs) 1-5 are indicated by red arrows and are listed in Table 2.
