# Supplementary figures and images for: Frequent Anti-V1V2 Responses Induced by HIV-DNA Followed by HIV-MVA with or without CN54rgp140/GLA-AF in Healthy African Volunteers
Source: Microorganisms. 2020 Nov 4;8(11):1722. doi: 10.3390/microorganisms8111722 (PMC7693996; doi:10.3390/microorganisms8111722)

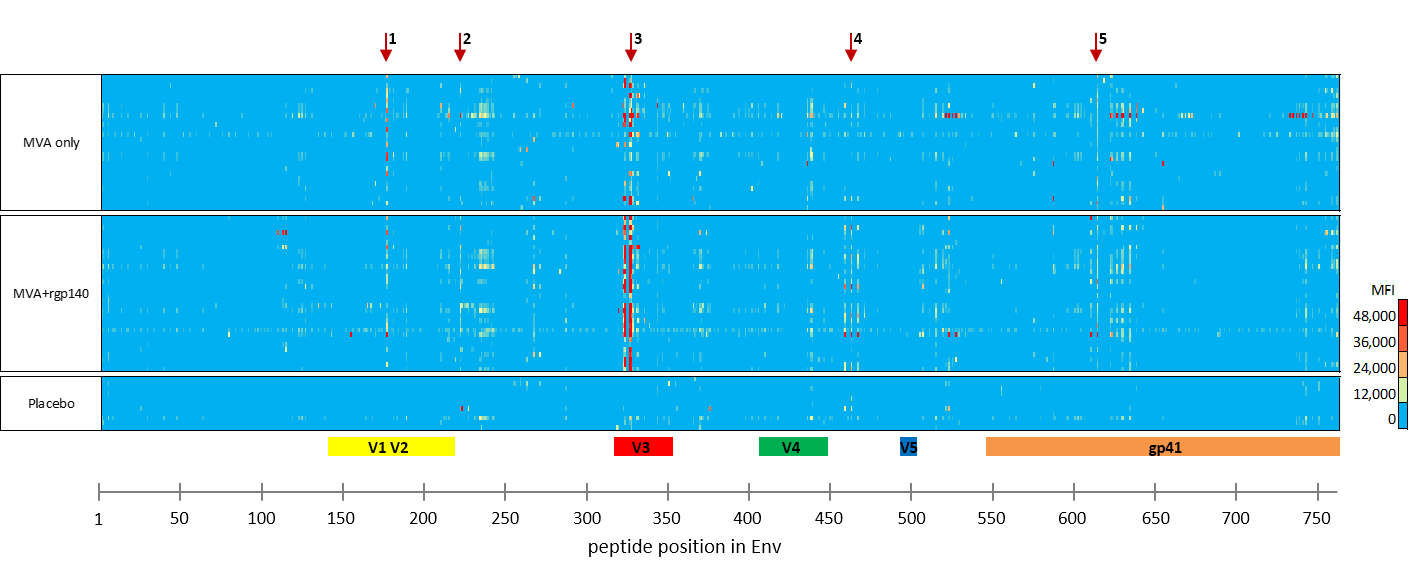

Supplement: Supplementary file 1 [file microorganisms-08-01722-s001.zip › Supplemetal materials/TaMoVacII_V1V2_FigS1.tif]
